# Supplementary material for: Improved measurement of disease progression in people living with early Parkinson’s disease using digital health technologies
Source: Commun Med (Lond). 2024 Mar 15;4:49. doi: 10.1038/s43856-024-00481-3 (PMC10942994; doi:10.1038/s43856-024-00481-3)
Supplement: Supplementary file 2 — Supplementary information [file 43856_2024_481_MOESM2_ESM.pdf]

SUPPLEMENTARY INFORMATION

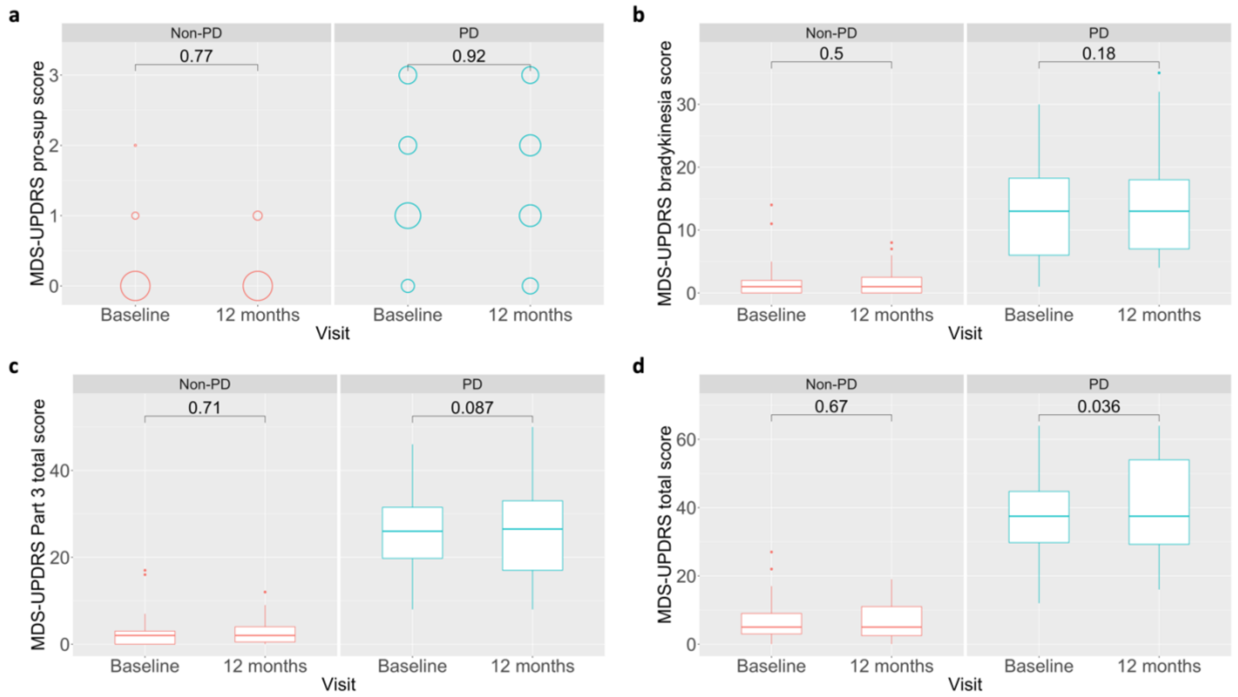

Supplementary Figure 1. MDS-UPDRS pronation-supination, bradykinesia, part 3 total, and total score sensitivity (paired Wilcoxon signed-rank test) to 1 year progression in PD patients. (a, b, c) MDS-UPDRS pronation-supination, bradykinesia, and part 3 total score do not significantly differ between baseline and 12-month visits in either PD or healthy participants, although part 3 total score (p=0.087) trends toward significance with PD patients. (d) MDS-UPDRS total score significantly distinguishes between baseline and 12-month visits in PD participants (p=0.036) but does not in healthy participants (p=0.67).

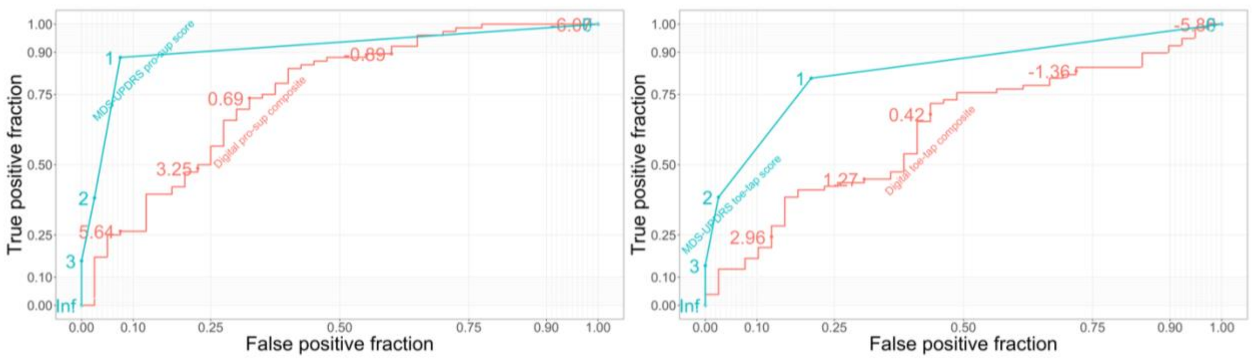

Supplementary Figure 2. ROC curve demonstrating known groups sensitivity of digital composite and MDS-UPDRS sub-scores for pronation-supination and toe-tapping for distinguishing PD from non-PD participants. Both pronation-supination (AUC=0.751) and toe-tapping (AUC=0.624) digital composites demonstrate moderate diagnostic discrimination. MDS-UPDRS pronation-supination (AUC=0.910) and toe-tapping (AUC=0.832) scores show good diagnostic sensitivity.

Digital feature/clinical score	Pronation-supination Non-PD BL vs. 12m	Pronation-supination PD BL vs. 12m	Toe-tapping Non-PD BL vs. 12m	Toe-tapping PD BL vs. 12m
Frequency (median)	0.073	0.4	↑0.0025	0.99
Frequency (var)	0.87	0.17	0.93	0.93
Frequency (slope)	0.067	0.53	0.58	↓0.03
Amplitude (median)	↑0.037	0.2	0.74	0.15
Amplitude (var)	0.95	0.14	0.46	0.5
Amplitude (slope)	0.22	↓0.0015	0.29	0.22
Max velocity (median)	↑0.011	0.48	↑0.04	0.055
Max velocity (var)	0.084	0.12	↓0.025	0.83
Max velocity (slope)	↓0.0028	↓0.001	0.15	0.7
Composite	0.9	↑0.018	↓0.011	↑0.011
MDS-UPDRS sub-score	0.77	0.92	0.77	0.62

p > 0.05; <= 0.05

Supplementary Table 1. Digital composite and individual feature sensitivity (paired Wilcoxon signed-rank test) to 1 year progression.

Digital feature/clinical score	Pronation-supination digital vs. clinical score	Pronation-supination PD vs. non-PD	Toe-tapping digital vs. clinical score	Toe-tapping PD vs. non-PD
Frequency (median)	0.21	0.024	0.17	0.017
Frequency (var)	0.15	0.35	0.78	0.26
Frequency (slope)	0.75	<0.001	0.75	0.12
Amplitude (median)	0.0057	<0.001	0.19	0.017
Amplitude (var)	0.99	0.16	0.016	0.0094
Amplitude (slope)	0.82	0.071	0.61	0.13
Max velocity (median)	0.037	<0.001	0.011	0.0058
Max velocity (var)	0.094	0.025	0.65	0.25
Max velocity (slope)	0.068	0.0025	0.19	0.018
Composite	0.028	<0.001	0.0011	0.029
MDS-UPDRS sub-score	NA	<0.001	NA	<0.001

$p > 0.05$; <= 0.05

Supplementary Table 2. Convergent (Kruskal-Wallis test) and known-groups (Wilcoxon signed-rank test) validity of digital composite and individual features and associated MDS-UPDRS sub-scores at baseline visit. One individual digital feature, median max velocity, significantly varied with clinical scores for both pronation-supination (Kruskal-Wallis p=0.037) and toe-tapping (Kruskal-Wallis p=0.011) (Supp. Table 2). Additionally, median amplitude varied significantly with clinical score for pronation-supination assessments (Kruskal-Wallis p=0.006), and variability of amplitude varied significantly with clinical score for toe-tapping assessments (Kruskal-Wallis p=0.011). Median frequency, median amplitude, median max velocity, and slope max velocity all significantly distinguished between PD and non-PD for both pronation-supination and toe-tapping assessments. Slope frequency and variability of max velocity significantly distinguished between PD and non-PD for pronation-supination, but not for toe-tapping. In contrast, variability of amplitude distinguished between PD and non-PD for toe-tapping, but not for pronation-supination.

Non-PD participant digital feature	Pronation-supination Baseline vs. 1 month	Pronation-supination 9 months vs. 12 months	Toe-tapping Baseline vs. 1 month	Toe-tapping 9 months vs. 12 months
Frequency (median)	0.67	0.82	0.6	0.78
Frequency (var)	0.29	0.69	0.47	0.39
Frequency (slope)	0.23	0.58	0.34	0.38
Amplitude (median)	0.38	0.75	0.73	0.67
Amplitude (var)	0.04	0.31	0.52	0.52
Amplitude (slope)	0.44	0.19	0.43	0.09
Max velocity (median)	0.71	0.87	0.46	0.46
Max velocity (var)	0.27	0.38	0.29	0.53
Max velocity (slope)	0.00	0.44	0.06	0.00
Digital composite	0.40	0.75	0.5	0.3

ICC <= 0.4; 0.4 to 0.74; >= 0.75

Supplementary Table 3. Test-retest reliability (intra-class correlation coefficients) of digital composites and individual features between visits in healthy participants. Between baseline and 1-month visits, individual digital features for pronation-supination (ICCs = 0-0.71) and toe-tapping (ICCs = 0.06-0.73) varied between good and poor test-retest reliability.

PD patient digital feature	Pronation-supination Baseline vs. 1 month	Pronation-supination 9 months vs. 12 months	Toe-tapping Baseline vs. 1 month	Toe-tapping 9 months vs. 12 months
Frequency (median)	0.62	0.68	0.65	0.66
Frequency (var)	0.34	0.59	0.46	0.54
Frequency (slope)	0.00	0.69	0.26	0.48
Amplitude (median)	0.65	0.58	0.49	0.71
Amplitude (var)	0.34	0.24	0.15	0.40
Amplitude (slope)	0.21	0.00	0.15	0.39
Max velocity (median)	0.64	0.74	0.51	0.33
Max velocity (var)	0.40	0.46	0.34	0.16
Max velocity (slope)	0.15	0.40	0.37	0.35
Digital composite	0.52	0.69	0.31	0.49

ICC <= 0.4; 0.4 to 0.74; >= 0.75

Supplementary Table 4. Test-retest reliability (intra-class correlation coefficients) of digital composites and individual features between visits in PD participants. Between baseline and 1-month visits, individual digital features for pronation-supination (0>ICC>0.65) and toe-tapping (0.15>ICC>0.65) varied between good to poor test-retest reliability.

Supplementary Note

```
########### Signal processing and peak detection

# second order Butterworth band-pass filter [0.3 20]
    fs=128; lowcut = 0.3; highcut = 20.0
    nyq = 0.5 * fs
    low = lowcut / nyq
    high = highcut / nyq
    sos = butter(2, [low, high], analog=False, btype='band', output='sos')
    df['eul_roll_bp'] = sosfiltfilt(sos, df['eul_roll'])

# spline interpolation with smoothing
    tck = interpolate.splrep(df['time'], df['eul_roll_bp'], s=len(df['time']))
    df['eul_roll_bp_ssi'] = interpolate.splev(df['time'], tck)

# peak detection
    maxima_eul = find_peaks(df['eul_roll_bp_ssi'])
    minima_eul = find_peaks(-df['eul_roll_bp_ssi'])

########### Feature extraction
# amplitude (difference in amplitude between peak to valley) of each movement is derived
# max velocity (maximum gyroscope amplitude between peak and valley) of each movement is
derived
# frequency (inverse of time between consecutive peaks) of each movement is derived
```

```
# median, standard deviation, and slope features are extracted for amplitude, max velocity, and frequency (ie. frequency feature extraction represented below)
    med_frequency = statistics.median(frequency_list)
    std_frequency = statistics.stdev(frequency_list)
    slope_frequency = np.median(frequency_list[-math.ceil(len(frequency_list)/3):]) - np.median(frequency_list[0:math.ceil(len(frequency_list) / 3)])
```
