# Supplementary material for: Improved measurement of disease progression in people living with early Parkinson’s disease using digital health technologies
Source: Commun Med (Lond). 2024 Mar 15;4:49. doi: 10.1038/s43856-024-00481-3 (PMC10942994; doi:10.1038/s43856-024-00481-3)
Supplement: Supplementary file 3 — Description of Additional Supplementary Files [file 43856_2024_481_MOESM3_ESM.pdf]

Description of Additional Supplementary Files

File Name: Supplementary Source Data
Description: Supplementary source data for figures 2, 3, 4 and 5.
