# Supplementary material for: Improved measurement of disease progression in people living with early Parkinson’s disease using digital health technologies
Source: Commun Med (Lond). 2024 Mar 15;4:49. doi: 10.1038/s43856-024-00481-3 (PMC10942994; doi:10.1038/s43856-024-00481-3)
Supplement: Supplementary file 5 — Reporting summary [file 43856_2024_481_MOESM5_ESM.pdf]

nature portfolio

Corresponding author(s): Matthew D. Czech

Last updated by author(s): Aug 28, 2023

Reporting Summary

Nature Portfolio wishes to improve the reproducibility of the work that we publish. This form provides structure for consistency and transparency in reporting. For further information on Nature Portfolio policies, see our Editorial Policies and the Editorial Policy Checklist.

Statistics

For all statistical analyses, confirm that the following items are present in the figure legend, table legend, main text, or Methods section.

n/a	Confirmed	
☐	☒	The exact sample size (n) for each experimental group/condition, given as a discrete number and unit of measurement
☐	☒	A statement on whether measurements were taken from distinct samples or whether the same sample was measured repeatedly
☐	☒	The statistical test(s) used AND whether they are one- or two-sided<br>*Only common tests should be described solely by name; describe more complex techniques in the Methods section.*
☒	☐	A description of all covariates tested
☒	☐	A description of any assumptions or corrections, such as tests of normality and adjustment for multiple comparisons
☐	☒	A full description of the statistical parameters including central tendency (e.g. means) or other basic estimates (e.g. regression coefficient) AND variation (e.g. standard deviation) or associated estimates of uncertainty (e.g. confidence intervals)
☐	☒	For null hypothesis testing, the test statistic (e.g. F, t, r) with confidence intervals, effect sizes, degrees of freedom and P value noted<br>*Give P values as exact values whenever suitable.*
☒	☐	For Bayesian analysis, information on the choice of priors and Markov chain Monte Carlo settings
☒	☐	For hierarchical and complex designs, identification of the appropriate level for tests and full reporting of outcomes
☐	☒	Estimates of effect sizes (e.g. Cohen's d, Pearson's r), indicating how they were calculated

Our web collection on statistics for biologists contains articles on many of the points above.

Software and code

Policy information about availability of computer code

Data collection	Raw wearable sensor data used in this study was collected using APDM Opal sensors.
Data analysis	Custom code was written using python v3.10 to process raw sensor data. Pseudocode is available within the supplementary section.

For manuscripts utilizing custom algorithms or software that are central to the research but not yet described in published literature, software must be made available to editors and reviewers. We strongly encourage code deposition in a community repository (e.g. GitHub). See the Nature Portfolio guidelines for submitting code & software for further information.

Data

Policy information about availability of data

All manuscripts must include a data availability statement. This statement should provide the following information, where applicable:

- Accession codes, unique identifiers, or web links for publicly available datasets
- A description of any restrictions on data availability
- For clinical datasets or third party data, please ensure that the statement adheres to our policy

The data are available to members of the Critical Path for Parkinson’s Consortium 3DT Initiative Stage 2. For those who are not a part of 3DT Stage 2, a proposal may be made to the WATCH-PD Steering Committee (via the corresponding author) for deidentified datasets.

Human research participants

Policy information about studies involving human research participants and Sex and Gender in Research.

Reporting on sex and gender	Information regarding participant sex was collected based on self-report during study enrollment, but information regarding gender was not. There were no planned sex or gender-specific analyses and no sex or gender-specific analyses were completed as part of the current report. All information regarding participant sex is included in disaggregated source data for this study.
Population characteristics	All participants recruited into this study were over 30yr of age. Participants recruited into the PD arm had to have a diagnosis of PD and be within two years of diagnosis. Participants recruited into the healthy comparison group could not have any diagnosed neurological or physical abnormalities based on self-report.
Recruitment	This was a multisite study and participants were recruited via a study website (https://watchpdstudy.org/), or flyers and word of mouth at individuals sites.
Ethics oversight	WCG Institutional Review Board approved the procedures used in the study

Note that full information on the approval of the study protocol must also be provided in the manuscript.

Field-specific reporting

Please select the one below that is the best fit for your research. If you are not sure, read the appropriate sections before making your selection.

☒ Life sciences ☐ Behavioural & social sciences ☐ Ecological, evolutionary & environmental sciences

For a reference copy of the document with all sections, see nature.com/documents/nr-reporting-summary-flat.pdf

Life sciences study design

All studies must disclose on these points even when the disclosure is negative.

Sample size	Given that this study was focused on measurement of disease progression in Parkinson's disease, and the lack of previous work using longitudinal digital measurement of the motor and non-motor features of PD, sample sizes were estimated using one-year clinical change on the MDS-UPDRS total score data from the Parkinson's Progression Marker Initiatve (PPMI) natural history study.
Data exclusions	Subject assessments with missing clinical scores, technical problems with data capture, or less than a threshold of 5 relevant movement repetitions detected during each recording session were excluded from analysis.
Replication	Given that this was a first-of-it's kind study designed to measure 12 month progression of PD using inertial sensor-based measures of bradykinesia, the focus of the current study was on algorithm development and description of results. However, the sensors used to complete the measurements have been used extensively in previous work and can constitute a 'kinematic gold standard' for movement assessment. In the current work, we also describe consistency of measures across multiple sessions (i.e., using ICC). Finally, these data are being independently analyzed as part of ongoing pre-competitive consortium work focused on replication and standardization of the measurements reported here.
Randomization	Given the case-control, observational nature of the study design presented here, participants were not randomized into the PD or neurologically healthy comparison groups
Blinding	As noted above, this was an observational study and blinding was not relevant.

Reporting for specific materials, systems and methods

We require information from authors about some types of materials, experimental systems and methods used in many studies. Here, indicate whether each material, system or method listed is relevant to your study. If you are not sure if a list item applies to your research, read the appropriate section before selecting a response.

Materials & experimental systems

n/a	Involved in the study
☒	☐ Antibodies
☒	☐ Eukaryotic cell lines
☒	☐ Palaeontology and archaeology
☒	☐ Animals and other organisms
☐	☒ Clinical data
☒	☐ Dual use research of concern

Methods

n/a	Involved in the study
☒	☐ ChIP-seq
☒	☐ Flow cytometry
☒	☐ MRI-based neuroimaging

Clinical data

Policy information about clinical studies

All manuscripts should comply with the ICMJE guidelines for publication of clinical research and a completed CONSORT checklist must be included with all submissions.

Clinical trial registration	NCT03681015
Study protocol	The full trial protocol is available from the investigators upon request
Data collection	This was a multisite observational study and all procedures were completed at 18 movement disorders clinics across the United States. Participants wore inertial sensors on each upper and lower limb while completing select bradykinesia prompts from the MDS-UPDRS Part 3 motor examination completed by trained movement disorders neurologists.
Outcomes	The primary outcomes of the study were the same as described in the current report: 1. Change and variability in inertial sensor-derived measures of motor function from baseline to 12 months during performance of the MDS-UPDRS part 3 motor exam.<br>2. Correlations between inertial sensor-derived measures of motor function and clinician ratings during performance of the MDS-UPDRS part 3 and total exam at each visit.
